# Capability of Utilizing *CYP3A5* Polymorphisms to Predict Therapeutic Dosage of Tacrolimus at Early Stage Post-Renal Transplantation

**DOI:** 10.3390/ijms16011840

**Published:** 2015-01-14

**Authors:** Takenori Niioka, Hideaki Kagaya, Mitsuru Saito, Takamitsu Inoue, Kazuyuki Numakura, Tomonori Habuchi, Shigeru Satoh, Masatomo Miura

**Affiliations:** 1 Department of Pharmacy, Akita University Hospital, 1-1-1 Hondo, Akita 010-8543, Japan; E-Mails: tniio-hki@umin.ac.jp (T.N.); hideaki-kagaya@hos.akita-u.ac.jp (H.K.); 2 Department of Urology, Akita University School of Medicine, Akita 010-8543, Japan; E-Mails: urosaito@gmail.com (M.S.); takmitz@gmail.com (T.I.); nqf38647@nifty.com (K.N.); thabuchi@gmail.com (T.H.); 3 Center for Kidney Disease and Transplantation, Akita University Hospital, Akita 010-8543, Japan; E-Mail: shigerus@doc.med.akita-u.ac.jp

**Keywords:** CYP3A5, tacrolimus, once-daily, dose variability, renal transplantation

## Abstract

While *CYP3A5* polymorphisms are used to predict the initial dosage of tacrolimus therapy, the predictive capability of genetic information for dosing at early stage post-renal transplantation is unknown. We investigated the influence of polymorphisms over time. An initial oral dose of modified-release once-daily tacrolimus formulation (0.20 mg/kg) was administered to 50 Japanese renal transplant patients every 24 h. Stepwise multiple linear regression analysis for tacrolimus dosing was performed each week to determine the effect of patient clinical characteristics. The dose-adjusted trough concentration was approximately 70% higher for patients with the CYP3A5*3/*3 than patients with the CYP3A5*1 allele before the second pre-transplantation tacrolimus dose (0.97 (0.78–1.17) *vs.* 0.59 (0.45–0.87) ng/mL/mg; *p* < 0.001). The contribution of genetic factors (CYP3A5*1 or *3) for tacrolimus dosing showed increased variation from Day 14 to Day 28 after transplantation: 7.2%, 18.4% and 19.5% on Days 14, 21 and 28, respectively. The influence of *CYP3A5* polymorphisms on the tacrolimus maintenance dosage became evident after Day 14 post-transplantation, although the tacrolimus dosage was determined based only on patient body weight for the first three days after surgery. Tacrolimus dosage starting with the initial administration should be individualized using the *CYP3A5* genotype information.

## 1. Introduction

Tacrolimus is an immunosuppressive agent used to prevent graft rejection after renal transplantation [1]. Because tacrolimus has a narrow therapeutic index, high intra- and inter-individual pharmacokinetic variability and a poor correlation between dosage and blood concentration, therapeutic drug monitoring (TDM) is an important tool for reducing the risk of nephrotoxicity and allograft rejection [1,2]. However, despite this drug monitoring effort, a large number of patients achieve trough concentrations that are above or below the targeted therapeutic range, particularly at the early stage post-transplantation [3,4,5].

Tacrolimus is a substrate of cytochrome P450 (CYP) 3A, and much of the inter-individual variability in its pharmacokinetics is explained by the presence of a single nucleotide polymorphism (SNP) in intron 3 of *CYP3A5* 6986A>G, resulting in the absence of a functional CYP3A5 protein in homozygous carriers (CYP3A5*3/*3; poor metabolizer, PM) [6,7,8]. The association between the *CYP3A5* genotype and tacrolimus pharmacokinetics is well established [9,10,11], and patients expressing functional CYP3A5 (carriers of the CYP3A5*1 allele; extensive metabolizer, EM), require approximately double the starting drug dose [12].

A modified-release once-daily (quaque die, QD) formulation (Advagraf in Europe and the USA, Prograf XL in Australia and Graceptor in Japan) has recently been developed to provide more convenient QD dosing that could improve patient adherence [13,14]. Recently, we reported that the pharmacokinetics of tacrolimus QD was strongly affected by the *CYP3A5* genotype in renal transplant patients [15,16]. Therefore, the narrow inter-individual variability of tacrolimus pharmacokinetics and its difference between *CYP3A5* EM and PM patients might contribute to a dosing strategy based on the *CYP3A5* genotype. However, the time period and degree to which the *CYP3A5* genetic information influences tacrolimus dosing at the early stage post-transplantation is unknown.

TDM is routinely used to individualize the dosage of tacrolimus [1,2]. Information about the *CYP3A5* genotype has not been used for a tacrolimus dosing strategy, although there is a considerable genetic basis to the inter-individual variability in tacrolimus pharmacokinetics. Therefore, knowing the pharmacokinetic and pharmacogenetic properties of tacrolimus and using *CYP3A5* genotype information for treatment individualization through TDM is crucial to improving treatment outcome. A drawback of TDM is that clinicians cannot adjust the dosage until the blood concentration is determined after administration of drug. On the other hand, *CYP3A5* genotyping could become a useful new tool for providing the optimal starting tacrolimus dosage for individual renal transplant patients.

While the *CYP3A5* polymorphism is predictive of the initial dosage of tacrolimus therapy prior to surgery, the predictive capability of genetic information at the early stage post-renal transplantation is unknown. We investigated the influence of the *CYP3A5* polymorphism over time, because the tacrolimus blood concentration reflects the clinical response. Our objective was to determine the contribution of the *CYP3A5* genotype on the temporal change of the tacrolimus maintenance dose at the early stage post-transplantation.

## 2. Results

Pre-transplant and post-transplant clinical characteristics of patients who received tacrolimus are listed in Table 1. The allele frequencies for CYP3A5*1 and *3 were 21.0% and 79.0%, respectively. None of the recipients had serious hepatic dysfunction. Hematocrit and serum albumin increased gradually after surgery.

**Table 1:** Clinical characteristics of renal transplant patients.

Items	Value	*p*-Value
Sex (males:females)	34:16	-
*CYP3A5* genotype groups		
*1/*1:*1/*3:*3/*3	4:13:33	-
Age (years, at transplantation)	50.5 ± 12.0 (21–70)	-
Body weight (kg)	57.9 ± 13.0 (34–91)	-
**Biochemical Data**		
Hematocrit (%) at pre-transplantation	30.3 (27.2–34.4)	-
on Day 7	28.8 (25.9–31.4)	<0.001
on Day 14	30.2 (28.0–33.2)	
on Day 21	31.4 (29.4–34.0)	
on Day 28	33.1 (29.8–35.2)	
Serum albumin (g/dL) at pre-transplantation	3.7 (3.5–4.0)	-
on Day 7	3.1 (2.8–3.4)	<0.001
on Day 14	3.6 (3.3–3.9)	
on Day 21	3.7 (3.5–3.9)	
on Day 28	3.9 (3.5–4.0)	
**Tacrolimus Data**		
Dose/day (mg) at pre-transplantation	12.0 (10.0–14.0)	-
on Day 7	12.0 (10.0–13.0)	<0.001
on Day 14	10.5 (9.0–13.0)	
on Day 21	9.0 (8.0–12.0)	
on Day 28	8.5 (6.0–11.0)	
*C*_24h_ (ng/mL) at pre-transplantation	10.6 (8.1–13.4)	-
*C*_0h_ on Day 7	11.0 (8.5–13.2)	<0.001
*C*_0h_ on Day 14	9.5 (7.9–12.4)	
*C*_0h_ on Day 21	8.2 (7.2–9.9)	
*C*_0h_ on Day 28	7.3 (6.6–8.0)	
Dose/body weight/day (mg/kg/day)	0.20 (initial oral dose in all patients)	-
on Day 7	0.21 (0.15–0.23)	<0.001
on Day 14	0.19 (0.12–0.23)	
on Day 21	0.16 (0.11–0.21)	
on Day 28	0.14 (0.10–0.21)	

Data presented as number, mean ± standard deviation (Range) or median (quartiles 1–3). *C*_0h_ or *C*_24h_, Concentration just prior to next dose.

**Figure 1:**
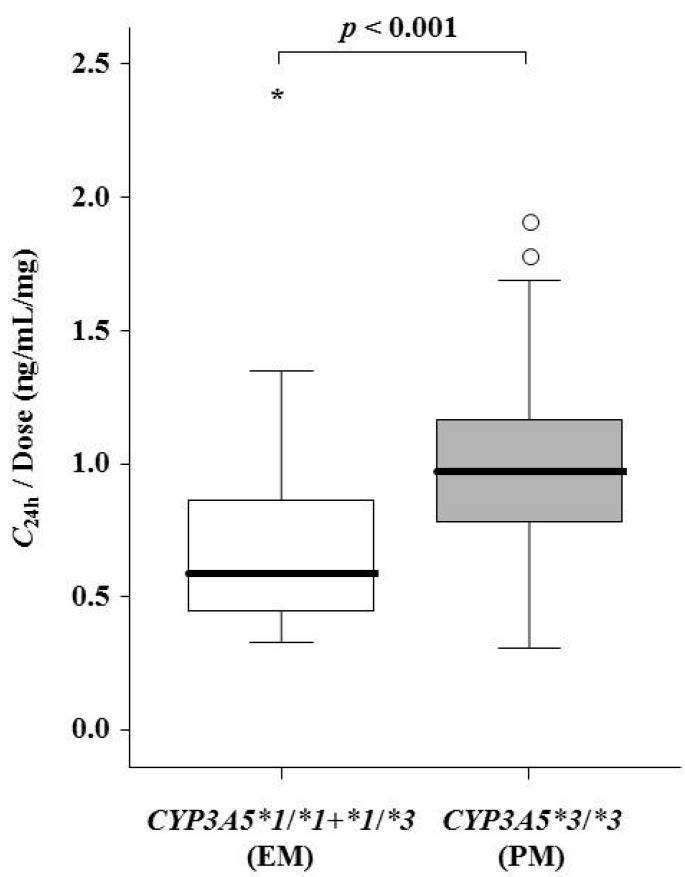
Comparison of dose-adjusted trough concentrations (*C*_24h_/dose) just prior to the second dose of tacrolimus pre-transplantation between *CYP3A5* extensive metabolizer (EM) (white boxes) and poor metabolizer (PM) (gray boxes) patients. Graphical analysis was performed using an SPSS box and whiskers plot. The box spans data between two quartiles (IQR), with the median represented as a bold horizontal line. The ends of the whiskers (vertical lines) represent the smallest and largest values that were not outliers. Outliers (circles) are values between 1.5 and 3 IQRs from the end of the box. Values of more than three IQRs from the end of the box are defined as extreme (asterisk).

The difference in the dose-adjusted *C*_24h_ between *CYP3A5* genotypes on the first day of tacrolimus administration pre-transplantation is shown in Figure 1. The dose-adjusted *C*_24h_ was approximately 70% higher for *CYP3A5* PM than *CYP3A5* EM patients (0.97 (0.78–1.17) *vs.* 0.59 (0.45–0.87) ng/mL/mg, *p* < 0.001).

The comparison and correlation with tacrolimus dosing on Days 7, 14, 21 and 28 after surgery and the clinical characteristics, laboratory test values or pharmacokinetics parameters on each day are listed in Table 2. There were no significant differences between tacrolimus dose and age, *C*_24h_ of tacrolimus pre-renal transplantation, *C*_0h_ or serum albumin on each day within a month after renal transplantation in this univariate analysis (Table 2).

**Table 2:** Comparison and correlation with tacrolimus daily dose on Days 7, 14, 21 and 28 post-transplantaion and clinical characteristics or pharmacokinetic parameters.

Items	Day 7		Day 14		Day 21		Day 28	
	Median (Quartiles 1–3)	*p*-Value	Median (Quartiles 1–3)	*p*-Value	Median (Quartiles 1–3)	*p*-Value	Median (Quartiles 1–3)	*p*-Value
Sex	-	0.001		0.096		0.660	-	0.409
males	12.5 (11.0–14.0)	-	12.0 (10.0–13.0)	-	9.0 (7.0–12.0)	-	8.0 (6.0–12.0)	-
females	10.0 (9.0–11.5)	-	9.0 (9.0–10.5)	-	9.0 (9.0–10.5)	-	9.0 (8.0–10.5)	-
*CYP3A5* genotype groups		0.629		0.363		0.010		0.010
*1/*1	12.5 (10.5–14.0)	-	12.5 (10.5–17.0)	-	12.5 (10.5–14.0)	-	11.5 (10.5–14.0)	-
*1/*3	12.0 (9.0–14.0)	-	12.0 (9.0–12.0)	-	10.0 (9.0–12.0)	-	10.0 (9.0–12.0)	-
*3/*3	12.0 (11.0–13.0)	-	10.0 (9.0–13.0)	-	8.0 (6.0–10.0)	-	7.0 (5.0–10.0)	-
	**Correlation Coefficient (*r*)**	***p*-Value**	**Correlation Coefficient (*r*)**	***p*-Value**	**Correlation Coefficient (*r*)**	***p*-Value**	**Correlation Coefficient (*r*)**	***p*-Value**
Age	−0.015	0.920	−0.076	0.602	−0.228	0.111	−0.168	0.244
Body-weight	0.704	<0.001	0.426	0.002	0.247	0.083	0.165	0.253
*C*_24h_ at pre-transplantation	0.061	0.674	−0.070	0.631	−0.023	0.876	−0.025	0.864
CL_t_ in CIV on day 3	0.382	0.006	0.549	<0.001	0.384	0.006	0.297	0.036
*C*_0h_ on the day	−0.170	0.238	−0.121	0.402	−0.174	0.226	−0.048	0.740
Hematocrit on the day	−0.084	0.563	−0.061	0.676	−0.231	0.106	−0.287	0.043
Serum albumin on the day	−0.184	0.201	−0.016	0.913	−0.159	0.270	−0.200	0.164

*C*_0h_ or *C*_24h_, concentration just prior to next dose; CL_t_ in CIV, tacrolimus clearance in 24h continuous intravenous infusion (CL_t_ = Daily dose/(Concentration on day 3 × 24)).

On the other hand, from the multiple regression analysis results, the tacrolimus dose was associated with the *CYP3A5* genotype, patient body weight, total clearance (CL_t_) of tacrolimus during continuous intravenous infusion (CIV) during the first three days after surgery and hematocrit on each day within the same time (Table 3). The overall *R*^2^ increased between the second and third weeks after surgery: 38.9%, 38.7%, 49.5% and 50.1% on Days 7, 14, 21, and 28, respectively.

**Table 3:** Stepwise selection multiple linear regression analysis of explanatory variable for tacrolimus daily dose after renal transplantation.

	Explanatory Variable	Slope	Bias *	SE *	*p*-Value *	*R*^2^
Day 7						0.389
	Body-weight (kg)	0.123	0.003	0.027	0.001	
	Intercept = 4.901		−0.128	1.500		
Day 14						0.387
	Body-weight (kg)	0.066	−0.003	0.031	0.033	
	CL_t_ in CIV on day 3 (L/h)	1.174	0.063	0.404	0.010	
	CYP3A5 *1/*1 (=1)	3.461	−0.019	1.494	0.011	
	Intercept = 2.695		0.009	1.746		
Day 21						0.495
	Body-weight (kg)	0.078	0.000	0.031	0.016	
	CL_t_ in CIV on day 3 (L/h)	0.885	0.026	0.346	0.012	
	CYP3A5 *3/*3 (=1)	−3.241	0.000	0.695	0.001	
	Hematocrit on the day (%)	−0.194	0.005	0.084	0.023	
	Intercept = 9.839		−0.201	2.946		
Day 28						0.501
	Body-weight (kg)	0.092	−0.001	0.035	0.019	
	CL_t_ in CIV on day 3 (L/h)	0.601	0.038	0.390	0.112	
	CYP3A5 *3/*3 (=1)	−3.747	0.025	0.720	0.001	
	Hematocrit on the day (%)	−0.284	0.011	0.071	0.002	
	Intercept = 12.889		−0.431	3.093		

*R*^2^, determination coefficient; SE, standard error; *C*_0h_ or *C*_24h_, concentration just prior to next dose; CL_t_ in CIV, tacrolimus clearance in 24 h continuous intravenous infusion (CL_t_ = Daily dose/(Concentration on day 3 × 24)). * calculated by using a bootstrap method.

**Figure 2:**
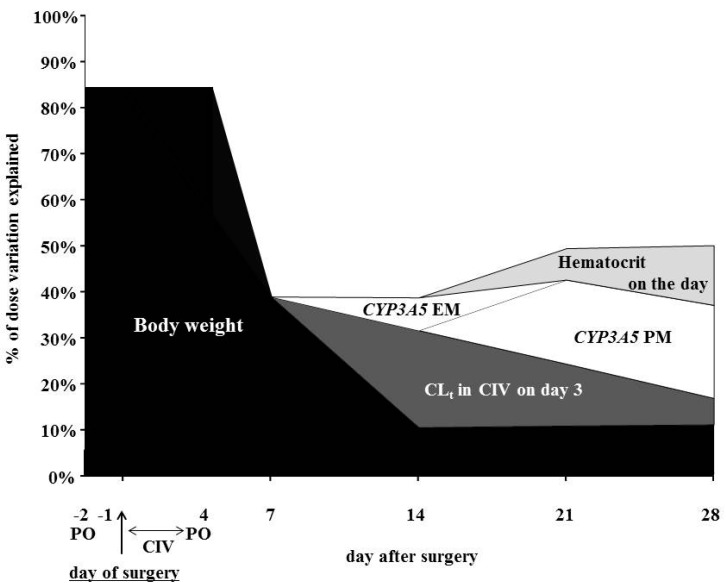
Percentage of dose variation for once-daily formulation of tacrolimus determined at weekly time points. CL_t_ during CIV; total clearance of tacrolimus during continuous intravenous infusion (CIV). PO; oral administration. EM, CYP3A5*1/*1 + *1/*3; PM, CYP3A5*3/*3.

The percentage of tacrolimus dose variation at weekly time points is shown in Figure 2. The contribution ratio of patient body weight for tacrolimus dosage gradually decreased over the initial two weeks and became constant thereafter: 85.0% on grafting operation day; 38.9% on Day 7 after transplantation; 10.3% on Day 14; 10.7% on Day 21; and 10.9% on Day 28. In contrast, the contribution ratio of genetic factors (CYP3A5*1 or *3) and hematocrit on each day for the tacrolimus daily dose increased from Day 14 to 28 after oral administration: 7.2% and 0% on Day 14; 18.4% and 6.9% on Day 21; and 20.4% and 13.1% on Day 28, respectively. On the other hand, the contribution ratio of CL_t_ of tacrolimus during CIV the first three days after surgery was maximum on Day 14 after surgery and gradually decreased thereafter.

**Figure 3:**
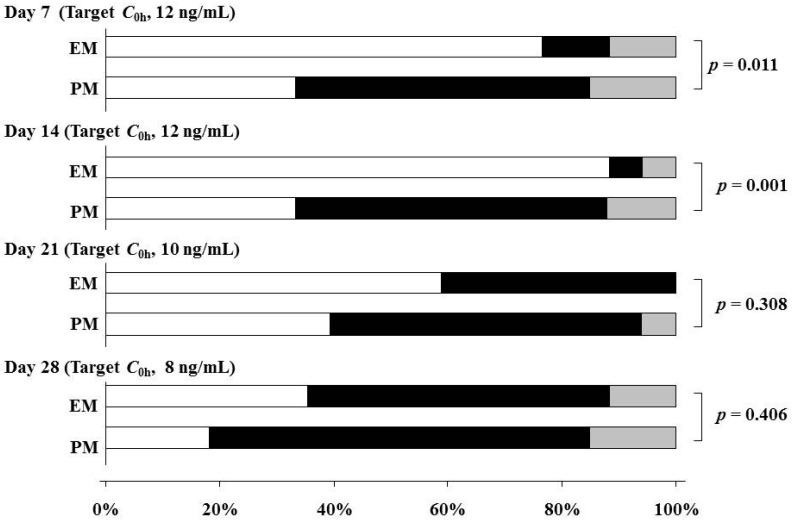
Comparison of achievement rates from the initial *C*_0h_ to the target range (target *C*_0h_ ± 20%) between *CYP3A5* genotypes at each stage after renal transplantation. White columns, lower than the target range; black columns, within the target range; gray columns; more than the target range. EM (*1/*1 + *1/*3), *n* = 17; PM (*3/*3), *n* = 33.

The achievement rates from the initial *C*_0h_ to the target range (target level ± 20%) were lower for *CYP3A5* EM than PM patients on Days 7 and 14 (11.8% *vs.* 51.5%, *p* = 0.011; and 5.9% *vs.* 54.5%, *p* = 0.001) (Figure 3). In particular, the rates lower than the target range on Days 7 and 14 were high in the *CYP3A5* EM group (76.5% and 88.2%). On the other hand, the achievement rates for the *CYP3A5* PM group were stable at more than 50% at the early stage post-transplantation (Figure 3).

**Figure 4:**
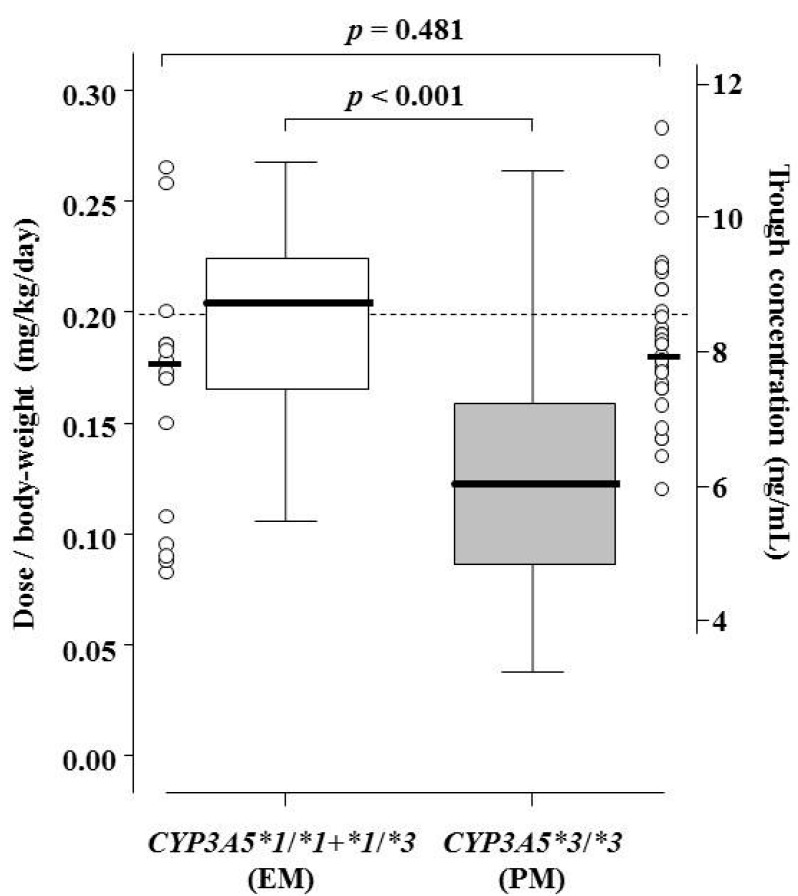
Comparison of body weight-adjusted dosage and trough concentration (open circles) of tacrolimus on Day 28 after surgery between *CYP3A5* EM and PM patients. Graphical analysis was performed using an SPSS box and whiskers plot. The box spans data between two quartiles (IQR), with the median represented as a bold horizontal line. The ends of the whiskers (vertical lines) represent the smallest and largest values that were not outliers.

The body weight-adjusted tacrolimus dose on Day 28 was approximately two-fold higher for *CYP3A5* EM than *CYP3A5* PM patients (0.21 (0.17–0.22) *vs*. 0.13 (0.09–0.16) mg/kg/day, *p* < 0.001), although there was no difference in *C*_0h_ at the same time (Figure 4).

In this population, none of the patients required a decrease or suspension of tacrolimus treatment at the early stage post-transplantation. On the other hand, the acute rejection rate was 23.5% and 6.0% in *CYP3A5* EM and PM patients, respectively (*p* = 0.093).

## 3. Discussion

The dose-adjusted *C*_24h_ on the first day of tacrolimus administration pre-transplantation was influenced by the *CYP3A5* polymorphism. In addition, the influence of the *CYP3A5* polymorphism on the tacrolimus maintenance dosage became evident after Day 14 post-transplantation, although the tacrolimus dosage was determined based only on patient body weight for the first three days after surgery. Therefore, the *CYP3A5* genotype information might influence the continuous tacrolimus dosing from the first day of tacrolimus administration pre-transplantation. Our present study shows that the initial dosage of tacrolimus should be decided based on the *CYP3A5* genotype.

The contribution ratio of patient body weight for tacrolimus dosage gradually decreased until Day 14 after switching to an oral agent on Day 4 after surgery. Therefore, it might be insufficient to consider only patient body weight to decide the dosage on the first day of tacrolimus administration. However, in the clinical setting, the initial dosage of tacrolimus is determined based only on patient body weight, and subsequently, the maintenance dosage of tacrolimus is adjusted by TDM based on trough concentrations [17]. In two studies using contemporary immunosuppressive regimens (tacrolimus, mycophenolate mofetil and steroid antibody induction), a lower trough concentration of tacrolimus in the first week post-transplantation is reported to be associated with a greater risk of acute rejection [18,19]. Therefore, it is important to predict an appropriate dose in the early stage post-transplantation. Although the body weight-adjusted initial dosage of tacrolimus was the same (0.20 mg/kg) for all patients, the dose-adjusted *C*_24h_ before the second dose was higher for *CYP3A5* PM than EM patients. Therefore, both *CYP3A5* genotype and patient body weight should be considered for optimal adjustment of the initial dosage of tacrolimus. *CYP3A5* EM patients could maintain a tacrolimus blood concentration at the same level as *CYP3A5* PM patients by increasing the initial dosage per body weight.

In the fourth week, the median of the body weight-adjusted tacrolimus dose for *CYP3A5* EM and PM patients was 0.21 and 0.13 mg/kg/day, respectively. On the other hand, the median of *C*_0h_ in both genotype groups was 8 ng/mL at the same phase. The dosage of tacrolimus required to achieve the target blood level varied considerably in renal transplant recipients. In our protocols, the blood trough target level within the second week of oral administration after CIV was 12 ng/mL. In addition, the majority of the *CYP3A5* EM group was not able to achieve the target range within Day 14 after tacrolimus administration post-transplantation. Therefore, the target of tacrolimus dosage starting with the initial administration in *CYP3A5* EM and PM patients is considered to be 0.30 and 0.20 mg/kg/day, respectively. Thervet *et al.* have reported that pharmacogenetic adaptation of the daily dose of Prograf, an oral, twice-daily formulation of tacrolimus, was associated with improved achievement of the target *C*_0h_ [20]. An individualized dosing regimen based on the *CYP3A5* genotype might be useful for shortening the time to reach the target *C*_0h_ of tacrolimus QD at the early stage post-transplantation.

The CL_t_ of tacrolimus during CIV the first three days after surgery influenced the change in tacrolimus dosage later than Day 7 after surgery. These data confirm that hepatic metabolism plays an important role in the pharmacokinetics of tacrolimus after oral administration. On the other hand, the dosage of tacrolimus appears to be influenced by the oral bioavailability of tacrolimus later than Day 14 after surgery. Because tacrolimus is typically released further along the gastrointestinal tract [6], *CYP3A5* expression in the intestinal tract has a greater influence on the pharmacokinetics of tacrolimus [16]. While the *CYP3A5* genotype is a factor in determining the dosage of tacrolimus, it is necessary to recognize that the optimal adjustment of dosage should be performed using both genotype and hepatic clearance at the early stage post-transplantation.

Hematocrit on each day was inversely correlated with tacrolimus dosage from Day 14 after surgery. The hematocrit values result in a fraction of tacrolimus accumulated in red blood cells. Many previous studies have reported that hematocrit values affected the blood concentration of tacrolimus with respect to intra- and inter-individual pharmacokinetic variability [21,22,23,24,25,26]. Therefore, we believe that hematocrit values play an important role in the pharmacokinetics of tacrolimus, *i.e.*, it might be necessary to take hematocrit into consideration for tacrolimus dosage regimens, when the hematocrit level changes significantly. However, in this study, approximately 10% of the patients had less than 25% hematocrit. The assay used in this study, the Architect-i1000^®^ system (Abbott Laboratories; Abbott Park, IL, USA), was reported to be free from hematocrit interference in the range 25%–55% [27]. The tacrolimus concentrations of patients with less than 25% hematocrit were detected to be higher than the actual values [28,29]. Therefore, we cannot rule out the possibility of a bias for low hematocrit values to tacrolimus dose adjustment.

There are still several limitations to this retrospective single-center study. First, the benefit of TDM for tacrolimus dosing using the *CYP3A5* genotype information has not been proven in this study. It is necessary to compare the optimal dosages with and without *CYP3A5* genotype information to definitively demonstrate the benefit of *CYP3A5* genetic analysis. Second, since the number of patients in this study was small, a comparison of the side effect rate directly attributable to tacrolimus and the acute rejection rate between *CYP3A5* genotypes was precluded. However, if the *CYP3A5* genotype is considered on the first day of tacrolimus administration pre-transplantation, the frequency of adjustment of tacrolimus dosage based on blood concentrations and total dose used at the early stage post-transplantation might decrease. Further studies must be conducted on the effect of tacrolimus TDM, including information about the *CYP3A5* genotype at the early stage post-transplantation.

## 4. Experimental Section

### 4.1. Patients and Protocols

This retrospective study of patients who were administered tacrolimus QD in renal transplantation enrolled 50 Japanese renal transplant patients who received renal grafts between November 2009 and October 2013. Most of the subjects in this study had participated in our previous studies [15,16]. The study protocol was approved by the Ethics Committee of Akita University School of Medicine, and all patients gave written informed consent. The patient eligibility criteria for the study were: (1) A tacrolimus-based immunosuppressive regimen, including mycophenolate mofetil (MMF; Cellcept^®^, Chugai Pharmaceutical, Tokyo, Japan), basiliximab and steroid; (2) an absence of pre-transplant donor-specific antibodies or delayed graft function; (3) no severe liver dysfunction or gastrointestinal motility; and (4) no introduction of drugs or foods that obviously affect CYP3A function during the study period. All patients received rabeprazole and the sulfamethoxazole-trimethoprim drug combination. Patients initially received a combination immunosuppressive therapy with tacrolimus and MMF starting 2 days prior to surgery. An initial oral dose of tacrolimus (0.20 mg/kg) and 1.5 g/day of MMF were given every 24 h at a designated time (09:00). Patients received a 24-h continuous intravenous infusion (CIV) of tacrolimus from the day of surgery. A CIV of 0.05 mg/kg per day of tacrolimus was administered for the first 3 days after surgery. On Day 3 after surgery, tacrolimus was changed from CIV to oral administration. The whole blood trough target level of tacrolimus was 15–20 ng/mL during CIV, 12 ng/mL within the second week post-transplantation, 10 ng/mL in the third week and less than 8 ng/mL thereafter. Meals were served at 7:30, 12:30 and 18:00 daily. Although the daily meal content (hospital diet which cooked chiefly polished rice and vegetables) varied for each patient, the amounts of energy, fat, protein and water were standardized (energy, 1700–2400 kcal; protein, 70–90 g; fat, 40–50 g; water, 1600–2000 mL), according to patient body weight. The renal transplant patients underwent an allograft biopsy on Day 28 after surgery. The Banff 97 classification and its updates were used to diagnose and categorize the lesions [30,31,32]. An acute rejection was diagnosed by two doctors based on the Banff criteria.

### 4.2. Sample Collection and Analytical Methods

On the first day of tacrolimus administration, whole blood samples were collected just prior to and 24 h after the morning dose. The following *C*_0h_ or *C*_24h_ showed blood concentrations of tacrolimus just prior to the next dose on Days 7, 14, 21 and 28 after surgery or just prior to the second dose pre-transplantation. Blood concentrations of tacrolimus were determined by the chemiluminescence magnetic microparticle immunoassay on the Architect-i1000^®^ system, according to the manufacturer’s instructions. The limit of detection of the Architect-i1000^®^ method was 0.25 ng/mL. Total imprecision was 7.0%, 5.7% and 4.8% at the three target concentrations of tacrolimus (3.2, 8.5 and 15.9 ng/mL), respectively. The method was linear up to 50 ng/mL. The limit of quantitation (LOQ) was 0.5 ng/mL.

### 4.3. Genotyping

DNA was extracted from a peripheral blood sample using a QIAamp Blood Kit (Qiagen, Hilden, Germany) and was stored at −80 °C until analyzed. For genotyping the CYP3A5*3 allele, the polymerase chain reaction-restriction fragment length polymorphism (PCR-RFLP) method was used [33]. The analysis results obtained from PCR-RFLP were confirmed using a fully automated single-nucleotide polymorphism (SNP) detection system (prototype i-densy^®^, ARKRAY Inc., Kyoto, Japan).

### 4.4. Pharmacokinetic Analysis

Pharmacokinetic parameters for tacrolimus were determined with a standard non-compartmental analytical method using WinNonlin (Version 5.2; Pharsight Co., Mountain View, CA, USA). The total clearance (CL_t_) of tacrolimus during the CIV on Days 1–3 was calculated using the formula below:

CL_t_ during CIV on Day 3 (L/h) = total dose per day (mg)/C_SS_ (ng/mL) × 24 (h)/1000; CL_t_ during CIV, total clearance of tacrolimus from the 24 h continuous intravenous infusion; C_SS_, steady-state concentration (on Day 3 after beginning 24-h CIV).

### 4.5. Statistical Procedures

In our previous studies [15,16], ten patients were the necessary sample size of one genotype to give α = 0.05 and β = 0.2 (power of 80%). To allow for stratification by genotype, more than 10 patients for each group were targeted for enrollment in this study. The Kolmogorov-Smirnov test was used to assess the distribution. The characteristics of renal transplant recipients were expressed as the mean ± standard deviation (range). The pharmacokinetic parameters for tacrolimus and laboratory test values on a day were expressed as medians (Quartiles 1–3). The chi-square test or Fisher’s exact test was used to examine the difference in categorical data, and the Kruskal-Wallis test or Mann-Whitney *U*-test was used to determine the difference in continuous values between groups. The Friedman test was used to determine the difference in continuous values within each patient. The Spearman’s rank correlation coefficient test was used to assess correlations with tacrolimus dose, and all results were expressed as a correlation coefficient of determination (*r*). Stepwise multiple linear regression analysis for tacrolimus dosing was performed to determine the effect of all factors examined in the univariate analysis. Backward-forward selection was used to select predictor variables and to quantify the corresponding partial determination coefficient (*R*^2^). For each subject, the *CYP3A5* genotypes were replaced with dummy variables (1 and 0, 0 and 1 and 0 and 0, respectively). The bias, standard error and *p*-value were calculated using the bootstrap method [34]. One thousand bootstrap samples were generated only once to reduce the variability of results for all regression analysis methods. A *p*-value less than 0.05 was considered statistically significant. Statistical analysis was performed with SPSS 20.0 for Windows (SPSS IBM Japan Inc., Tokyo, Japan).

## 5. Conclusions

In conclusion, with an initial daily dose of 0.20 mg/kg of tacrolimus, the *C*_24h_ before the second dose was higher for *CYP3A5* PM than *CYP3A5* EM patients. In addition, the influence of the *CYP3A5* polymorphism on the tacrolimus maintenance dosage became evident after Day 14 post-transplantation, although the tacrolimus dosage was determined based only on patient body weight the first three days after surgery. Individualization of the initial tacrolimus dosage might be possible using *CYP3A5* genotype information.
